# Diagnostic value of neutrophil to lymphocyte ratio in differentiation of ruptured ovarian cysts and adnexal torsion

**DOI:** 10.4274/tjod.95881

**Published:** 2018-06-21

**Authors:** Sunullah Soysal, Rezzan Berna Baki

**Affiliations:** 1 Marmara University Faculty of Medicine, Department of Obstetrics and Gynecology, İstanbul Turkey

**Keywords:** Adnexal torsion, ovarian cyst, rupture, neutrophil, lymphocyte

## Abstract

**Objective:**

Ovarian cyst rupture and adnexal torsion (AT) differential diagnosis is important for early surgical intervention of AT for preserving ovarian function. The aim of this study was to evaluate the diagnostic value of preoperative the neutrophil-to-lymphocyte ratio (NLR) in patients with adnexal torsion and ovarian cyst rupture.

**Materials and Methods:**

Data of 80 patients who underwent surgery between 2012 and 2017 for ovarian cyst rupture, adnexal torsion, and unruptured ovarian cyst were analyzed. Patients were categorized as adnexal torsion (n=35), ovarian cyst rupture (n=20), unruptured ovarian cyst (n=25) groups. Preoperative NLR were compared among the three groups of the patients.

**Results:**

The adnexal torsion group had a median NLR of 8.0 (range, 4.0-14.1), the ovarian cyst rupture group had a median of NLR 7.5 (range, 3.7-11.5), and median NLR of the unruptured ovarian cyst group was 2.2 (range,1.8-2.7). The NLR was found to have a difference that reached statistical significance among the three groups (p<0.001). When the groups were individually compared, there was no significant difference between the ovarian cyst rupture and adnexal torsion groups (p=0.372), but there was a significant difference between the unruptured ovarian cyst and adnexal torsion groups (p<0.001).

**Conclusion:**

NLR may be useful in the differential diagnosis of unruptured ovarian cyst from adnexal torsion, but it has no diagnostic value for the differentiation of ovarian cyst rupture and adnexal torsion.

**PRECIS:** Neutrophil to lymphocyte ratio can be used in preoperative differential diagnosis of ovarian cysts and adnexal torsion. But the diagnostic value of Neutrophil to lymphocyte ratio in differentiation of ruptured ovarian cyst from adnexal torsion cases was not determined in this study.

## Introduction

Adnexal torsion (AT) can be defined as total or partial rotation of the adnexa around its own vascular axis. It results in venous and lymphatic blockage of ovarian tissue and causes congestion and hemorrhagic infarction leading to gangrene and hemorrhagic necrosis^(1)^. AT cases are generally seen in the reproductive age group^(2)^. Although AT has the highest incidence among women aged between 20 and 30 years, it can be experienced by women at any age. It is diagnosed in 2-7% women who undergo surgery for acute pelvic pain^(3)^.

The diagnosis of AT is important for preserving fertility. Early diagnosis and detorsion of the adnexa may preserve ovarian function and fertility. Although conventional methods are used for the diagnosis of AT, only 46% of patients who are diagnosed as having AT have real torsion during surgery and no specific diagnostic modality has yet been defined for the detection of AT^(4)^. The white blood cell (WBC) count increases during inflammatory processes in the body. It was shown that WBC count increases in cases of AT^(5)^. Many studies have shown that the neutrophil-to-lymphocyte ratio (NLR) is a significant inflammatory marker in various diseases^(6,7)^. The mean platelet (PLT) volume (MPV) is also important for the diagnosis of inflammatory states. The diagnostic importance of MPV has been shown in diseases such as acute appendicitis, pelvic inflammatory, and ectopic pregnancy^(7,8,9)^. Patients with pelvic pain due to adnexal causes should be diagnosed carefully. Ruptured or unruptured ovarian cysts (UOC) may cause pelvic pain. Clinical conditions requiring surgery should be differentiated from follow-up patients. The aim of this study was to investigate the diagnostic value of the NLR in the differential diagnosis of AT with ruptured or UOC.

## Materials and Methods

### Patients and data

A database of the 80 patients who underwent surgery for AT and ovarian cysts between January 2012 and June 2017 was retrospectively investigated. Patients with a suspicion of malignancy and tubal ovarian abscess were excluded from the study because malignancies and abscess formation may have an effect on blood count parameters. Three groups were formed [AT (n=35), UOC (n=25), and ovarian cyst rupture (OCR) (n=20)] and investigated. Demographic features, WBCs, neutrophil, lymphocyte, and PLT counts and hemoglobin (Hb) levels, NLR, MPV and red cell distribution width (RDW)  before surgery were recorded. This study was approved by the Iocal Research Ethics Committee of Marmara University Faculty of Medicine the İnstitution (date: 09.2017 approval number: 543).

### Statistical Analysis

Statistical analyses were performed using the SPSS 20.0 (SPSS, Version 20.0; Chicago, IL, USA) statistics software. In the study, descriptive and categorical data were evaluated as number (n) and percentage (%), and continuous data were studied as interquartile range and medians. The Mann-Whitney U test and Kruskal Wallis test were used for comparisons. The significance level was accepted as p<0.05.

## Results

### Demographics

The demographic findings of the groups were evaluated (Table 1). The median ages of the AT OCR, and UOC groups were 24, 32.5, and 34 years, respectively. There was a significant difference in respect to age and parity status of the patients. This difference was an expected feature of the AT cases, which are mostly seen at younger ages.

### Complete Blood Counts

The median WBC count for the AT group was 12.1x10^3^/µL, OCR was 12.0x10^3^/µL, and the UOC was 7.3x10^3^/µL. There was a statistically significant difference between the groups (p<0.001). When the AT group was compared with the other groups individually, the AT group had no significant difference with the OCR group but had a significant difference with the UOC group (p=0.0834 and p<0.001).  The median Hb levels of the AT, OCR, and UOC groups were 11.6 g/dL, 12.0 g/dL, and 12.5 g/dL (p<0.001). When further analysis was conducted, the statistically significant difference was due to the difference between the AT group and the UOC (p<0.001); no significant difference was noted between the AT and OCR groups. The median neutrophil counts for groups were found as 9.9x10^3^/µL for the AT group, 9.9x10^3^/µL for the OCR group, and 4.7x10^3^/µL for the UOC group. The significant difference was attributable to the difference between the AT group and UOC group, rather than the difference between the AT and OCR groups (p<0.001 and p=0.986). The median lymphocyte count was 1.3x10^3^/µL for the AT group, 1.4x10^3^/µL for the OCR group, and 2.0x10^3^/µL for the UOC group. When the AT group was compared with the OCR group, no statistically significant difference was found regarding lymphocyte counts (p=0.273), whereas there was a statistically significant difference between the AT and the UOC groups (p=0.009). The median RDWs, PLTs, and MPVs for all groups showed no statistically significant difference. The NLR was found to have a difference that reached statistical significance (p<0.001). When the groups were individually compared with the AT group, NLR was not found to be different to the OCR group (p=0.372), but there were a significant difference with the UOC group (p<0.001). Table 2 shows the complete blood count results and NLR of the three groups.

## Discussion

AT has a frequency of 2.5 to 7.4% in emergency gynecologic operations. It is common in reproductive-age women and early diagnosis of this condition is vital for the preservation of the vitality of the ovary^(10)^. AT has nonspecific symptoms such as aches, nausea, vomiting, and low-grade fever, and as such, it is difficult to diagnose. OCR, ectopic pregnancy, adnexitis, acute appendicitis, gastroenteritis, and renal colic should be differentiated from AT^(11)^. The diagnosis of AT is generally based on ultrasonography. An increase in ovarian size, cyst of the adnexa, and free liquid in the pelvic cavity are the findings in ultrasound. However, ultrasound was shown to be normal in nearly half of all AT cases, especially in children^(12)^. Another diagnostic method is color doppler ultrasound, which is widely used for diagnosis of AT; doppler flow absence or decrease in ultrasound favors the diagnosis of AT. The detection of a normal blood flow pattern due to the dual blood supply of ovaries in 60% of cases of AT makes the role of doppler ultrasound in preoperative diagnosis debatable^(13,14)^.

NLR has been a popular parameter for the diagnosis of several inflammatory and surgical conditions in which it has been shown to be superior to WBC counts^(15)^. NLR has also been shown to be useful in the diagnosis of many diseases concerning gynecologic inflammatory disorders and malignancies in several studies^(16,17,18)^.

Pelvic pain requiring surgery due to adnexal pathologies may sometimes be a confusing issue for surgeons, especially in cases of OCR, which can be clinically followed up without a surgical intervention. Therefore, we aimed to investigate the role of NLR for the differential diagnosis of AT from OCR and UOC in this study.

Ercan et al.^(19)^ compared preoperative NLR and WBC counts in patients with AT and ovarian cysts. They found an increase in both WBC and neutrophil counts in both groups, but a lower lymphocyte count in the ovarian cyst group. NLR was found to be significantly higher in the AT group compared with the ovarian cyst group^(19)^. We also found a significant difference in both WBC and neutrophil and lymphocyte counts and NLR of AT, OCR, and UOC. When the groups were individually compared, it was found that there was no significant difference between the OCR group and the AT group, whereas a significant difference was found between the AT group and the UOC group. In a similar study, AT and ovarian cyst groups were compared with several complete blood count markers. NLR, WBC, and neutrophil counts were found to be higher in the AT group compared with the ovarian cyst group. We also found the same difference, but as above, it was due to the UOC and AT group difference rather than the UOC and AT group difference. MPV and platelet counts were also investigated in the same study and no difference was found between the groups, similar to our findings^(20)^. OCR may need surgical intervention but can also be followed up clinically in hemodynamically stable cases. Observation with analgesia can usually be used for the management of hemorrhagic cysts and cyst rupture. Surgery should be performed in cases in which there is hemodynamic compromise, diagnostic uncertainty or suspicion of torsion, no relief of symptoms within 48 hours, and an increase in hemoperitoneum, and a decrease in HB level is detected^(21)^. Ruptured cysts and AT are different in respect to management, clinical follow-up of a ruptured cyst may be useful in specific cases, but early surgical intervention is important for preserving fertility in AT. NLR has been shown to be useful in differentiating ovarian cysts and AT^(19,20)^. Contrary to those findings, we found that NLR was not useful in the differential diagnosis of OCR and AT cases.

### Study Limitations

The retrospective nature of the study is a limiting factor. Only data of patients of a single center were collected. Number of cases should be increased for to reach a clearer conclusion about the diagnostic value of neutrophil to lymphocyte ratio in differential diagnosis of adnexal torsion and ovarian cysts.

## Conclusion

NLR can be used in the preoperative differential diagnosis of ovarian cysts and AT. However, the diagnostic value of NLR in the differentiation of OCR and AT cases was not determined in this study.

## Figures and Tables

**Table 1:**
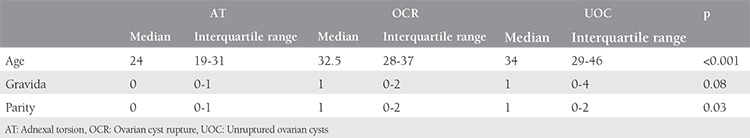
Demographic findings

**Table 2:**
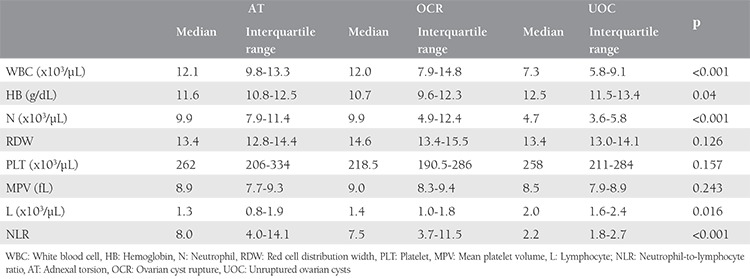
Complete blood count parameters and neutrophil-to-lymphocyte ratio
